# The association of prescription opioid use with suicide attempts: An analysis of statewide medical claims data

**DOI:** 10.1371/journal.pone.0269809

**Published:** 2022-06-30

**Authors:** Chongliang Luo, Kun Chen, Riddhi Doshi, Nathaniel Rickles, Yong Chen, Harold Schwartz, Robert H. Aseltine

**Affiliations:** 1 Department of Biostatistics, Epidemiology and Informatics, Perelman School of Medicine, University of Pennsylvania, Philadelphia, PA, United States of America; 2 Division of Public Health Sciences, Washington University School of Medicine in St. Louis, St Louis, MO, United States of America; 3 Department of Statistics, University of Connecticut, Storrs, CT, United States of America; 4 Center for Population Health, Uconn Health, Farmington, CT, United States of America; 5 Beacon Health Options, Rocky Hill, CT, United States of America; 6 Department of Pharmacy Practice, School of Pharmacy, University of Connecticut, Storrs, CT, United States of America; 7 Institute of Living, Hartford Healthcare, Hartford, CT, United States of America; 8 Department of Psychiatry, University of Connecticut Health Center, Farmington, CT, United States of America; 9 Division of Behavioral Sciences and Community Health, Uconn Health, Farmington, CT, United States of America; Universita degli Studi di Milano-Bicocca, ITALY

## Abstract

**Background:**

Suicides and opioid overdose deaths are among the most pressing public health concerns in the US. However direct evidence for the association between opioid use and suicidal behavior is limited. The objective of this article is to examine the association between frequency and dose of prescription opioid use and subsequent suicide attempts.

**Methods and findings:**

This retrospective cohort study analyzed 4 years of statewide medical claims data from the Connecticut All-Payer Claims Database. Commercially insured adult patients in Connecticut (n = 842,773) who had any medical claims beginning in January 2012 were followed through December 2015. The primary outcome was suicide attempt identified using International Classification of Diseases (ICD 9) diagnosis codes. Primary predictor variables included frequency of opioid use, which was defined as the number of months with claims for prescription opioids per year, and strength of opioid dose, which was standardized using morphine milligram equivalent (MME) units. We also controlled for psychiatric and medical comorbidities using ICD 9 codes. We used Cox proportional hazards regression to examine the association between frequency, dose, and suicide attempts, adjusting for medical and psychiatric comorbid conditions. Interactions among measures of opioid use and comorbid conditions were analyzed.

In this cohort study with follow-up time up to 4 years (range = 2–48 months, median = 46 months), the hazard ratios (HR) from the time-to-event analysis indicated that patients prescribed opioid medications for at least 6 months during the past year and at 20–50 MME levels or higher had 4.44 (95% CI: [3.71, 5.32]) to 7.23 (95% CI: [6.22, 8.41]) times the risk of attempted suicide compared to those not prescribed opioids. Risk of suicide attempt was sharply elevated among patients with psychiatric conditions other than anxiety who were prescribed more frequent and higher opioid doses. In contrast, more frequent and higher doses of prescription opioids were associated with lower risk of suicide attempts among patients with medical conditions necessitating pain management.

This study is limited by its exclusive focus on commercially insured patients and does not include patients covered by public insurance. It is also limited to patients’ receipt of prescription opioids and does not take into account opioids obtained through other means, nor does it include measures of actual patient opioid use.

**Conclusions:**

This analysis provides evidence of a complex relationship among prescription opioids, mental health, pain and other medical comorbidities, and suicide risk. Findings indicate the need for proactive suicide surveillance among individuals diagnosed with affective or psychotic disorders who are receiving frequent and high doses of opioids. However, appropriate opioid treatment may have significant value in reducing suicide risk for those without psychiatric comorbidities.

## Introduction

Deaths associated with prescription opioid use have been among the most pressing public health concerns of the past decade [1–3]. Data from the CDC indicates that overdose deaths in the US involving prescription opioids increased to 16,416 in 2020, reversing a two-year downward trend from a peak of 17,000 deaths in 2017, and that one quarter of all opioid overdose deaths involved prescription opioids [4]. Despite a decline in the overall opioid prescribing rate in the US over the last 8 years, prescribing rates continue to remain very high in certain areas of the country [5].

During this same period the US has experienced an unprecedented increase in suicide rates. According to the CDC, the annual suicide rate in the US increased 33% from 1999 to 2019 [6]. A recent study of suicidal drug overdoses from 11 US states indicated that opioids account for approximately 40% of fatal suicide poisonings [7]. There appears to be a complex, bidirectional association between opioid use and depression, the major proximate risk factor for suicidal behavior [8]. Evidence suggests that use of opioid analgesics for longer than 30 days and with a rapidly escalating dose conveys a risk of new-onset depression [9,10]. However, a survey of 1,334 patients on chronic opioid therapy for noncancer pain found that patients with both moderate and severe depression were substantially more likely to misuse their opioid medications for non–pain symptoms [11]. A recent review article summarized this bidirectional association: the literature indicates that depressed patients were more likely to initiate opioid therapy and substantially more likely to transition to long term use than non-depressed patients, while long-term opioid therapy increases the risk of incident, recurrent, and treatment-resistant depression [12].

However, direct evidence for the association between opioid use and suicidal behavior is limited. Studies using data from US national surveys revealed that self-reported prescription opioid misuse, as well as frequent or persistent use of opioid medications, were significantly associated with suicide ideation and self-reported attempts [13,14]. Clinical evidence of this association was found in a recently published suicide risk algorithm, where opioid use disorder emerged as a predictor of medically treated suicide attempts in large patient samples [15]. There is also robust evidence of the association between discontinuation or dose tapering of prescription opioids and increased risk of mental health crises or suicidal behavior among both US veterans and general patient populations [16–18].

This study addresses the limitations in this literature by examining the impact of both the frequency and dose of prescription opioids on suicide attempts among commercially insured adults in Connecticut. Data were obtained from the Connecticut All-Payer Claims Database (APCD), which aggregates medical and pharmacy claims across healthcare providers and care settings [19]. This feature of an APCD addresses the gaps and fragmentation associated with clinical data derived from a single health system or provider network [20], which is especially important with frequent users of prescription opioids, whose complex medical histories and treatment patterns are likely to yield an incomplete picture of patients when derived from more limited clinical datasets.

## Methods

This study was approved by the Data Release Committee of the Connecticut APCD and was determined to be non-human subjects research by the UConn Health Institutional Review Board.

### Study design and measures

Medical and pharmacy claims from all fully insured commercial beneficiaries (N = 842,773) aged 18–65 were obtained from the Connecticut APCD. The study population consisted of patients who had any claims during a recruiting window from January 1, 2012 to December 31, 2013, who were followed until either a first suicide attempt diagnosis or the conclusion of the observation period (December 31, 2015). Patients whose first recorded visit was a suicide attempt were excluded due to the lack of prior information, as were patients with a diagnosis of opioid use disorder (S2 Table).

Suicide attempts were identified using ICD-9 diagnostic codes. Following recent research showing the limitations of ICD-9 external cause of injury codes (E-codes) in claims data, suicide attempts were identified by both E-codes and other ICD-9 code combinations indicative of suicidal behavior (e.g., injury with suicidal ideation) [21–23]. S1 Table provides the algorithms for suicide attempt identification.

Opioid prescriptions were identified from pharmacy claims by matching National Drug Codes to the CDC’s opioid drug dictionary [24]. All pharmacy claims, including those paid for by the patient, were included. Medications used to treat opioid use disorder such as buprenorphine (and its combinations except those used to treat moderate or severe chronic pain), naltrexone (including morphine/naltrexone combinations), and methadone were excluded from the analysis.

Patterns of opioid use examined in the study population included an analysis of the frequency and dose during the last 6 months of the observation period compared to prior use. Fr*equency of opioid use* was defined as the number of months with claims for prescription opioids per year. Patients’ *opioid dose* was standardized using the morphine milligram equivalents (MME) per day measure. The MME per day was calculated as: [25]

MME/day=strengthperunit×(quantity/dayssupply)×MMEconversionfactor

Frequency was categorized into four levels including no use, low (0–1 month/year), medium (1–6 months/year) and high (6–12 months/year). Dose was categorized into five levels including no use, low (0–20 MME/day), medium (20–50 MME/day), high (50–100 MME/day), and very high (>100 MME / day). Frequency and dose were jointly categorized into 13 categories, with “no use” used as the reference group. We elected to use this coding approach for three reasons. First, we observed a nonlinear association between the frequency of use and suicide attempts in preliminary analysis, which argued against treating frequency as a continuous variable. Second, we observed a statistical interaction involving dose and frequency in the prediction of suicide attempts, necessitating the interpretation of each variable contingent on the level or value of the other. Third, this joint categorization enables further analysis of the interaction between opioid usage and comorbidity conditions without involving 3-way interaction terms. S1 Fig provides an illustration of the study design and explains how the frequency and dose of opioid usage were calculated.

Medical and psychiatric conditions found in the literature to be related to opioid use and suicidal behavior were also included in the analysis [8,15,26,27]. These include headache, neuropathy, chronic pain, acute pain, cancer, depression and bipolar disorder, psychotic disorder, substance use disorder (excluding opioid use disorder), post-traumatic stress disorder (PTSD), and anxiety, and were measured during the recruiting window. The ICD 9 diagnosis codes and code combinations for each condition presented in S2 Table. The patient’s age and sex were included as covariates in the analyses; race and ethnicity are typically not collected by commercial insurers and was not available in the APCD [28].

### Statistical analyses

We used multivariable Cox regression to examine the association of frequency and dose in prescription opioid use with time to suicide attempts, while adjusting for age, sex and medical/psychiatric conditions [29]. We further examined the influence of medical and psychiatric conditions on the association between prescription opioid use and suicide attempts by including 2-way interaction terms in this model. Predictive performance of the Cox regression model was measured by randomly splitting the study population into 80% training set and 20% testing set for model validation. Suicide attempts at one year after follow-up among patients in the testing set were predicted by their calculated probability using the trained model. We repeated the random splitting 200 times and computed area under the curve (AUC) and receiver operating characteristic (ROC) curves by comparing the predicted and actual outcomes for the patients in the testing set. To measure the contribution of opioid use to the prediction of suicide attempts, nested models were fitted with or without the opioid use variables and compared the AUC values. A significance level of 0.05 was used in each statistical test and incorporated multiple testing adjustments using Bonferroni corrections when testing interactions between opioid use and patient comorbidities, i.e., an interaction term with p-value < 0.0004 was significant. All analyses were performed using R software [30].

## Results

Table 1 provides a description of the study population. Among the 842,773 patients with a medical claim during the recruiting window, 294,534 (34.9%) had been prescribed opioids, with the most common prescribing pattern being a single prescription per year within the observation period (26.9%) at a medium (20–50 MME/day) dose (21.0%). A total of 4,504 (0.53%) patients had a suicide attempt during the observation period.

**Table 1:** Demographic characteristics and medical and psychiatric conditions among the study population (N = 842,773).

Variables	Suicide Attempter		Non-Attempter		Total	
	N	%	N	%	N	%
**Totals**	4,504	100	838,269	100	842,773	100
**Sex**						
Women	2,584	57.4	454,001	54.2	456,585	54.2
Men	1,920	42.6	384,268	45.8	386,188	45.8
**Age category (years)**						
18–25	670	14.9	129,044	15.4	129,714	15.4
26–39	832	18.5	181,990	21.7	182,822	21.7
40–54	3002	66.7	527,235	62.9	530,237	62.9
**Medical conditions**						
Headache	876	19.4	89,148	10.6	90,024	10.7
Neuropathy	245	5.4	32,564	3.9	32,809	3.9
Chronic pain	2,805	62.3	456,158	54.4	458,963	54.5
Acute pain	2,394	53.2	362,243	43.2	364,637	43.3
Cancer	841	18.7	257,577	30.7	258,418	30.7
**Psychiatric conditions**						
Depression / bipolar	2,731	60.6	109,270	13.0	112,001	13.3
Psychotic	901	20.0	14,462	1.7	15,363	1.8
Substance	971	21.6	21,229	2.5	22,200	2.6
PTSD	290	6.4	8,312	1.0	8,602	1.0
Anxiety	977	21.7	83,387	9.9	84,364	10.0
**Opioid use** *						
no use	2,121	47.1	546,118	65.1	548,239	65.1
frequency low	507	11.3	225,848	26.9	226,355	26.9
frequency medium	1006	22.3	47,880	5.7	48,886	5.8
frequency high	870	19.3	18,423	2.2	19,293	2.3
dose low	418	9.3	46,799	5.6	41,586	5.6
dose medium	1,286	28.6	175,448	20.9	165,488	21.0
dose high	452	10.0	59,102	7.1	60,750	7.1
dose very high	227	5.0	10,802	1.3	11,345	1.3

* Opioid use frequency is categorized as low (0–1 month/year), medium (1–6 months/year), and high (6–12 months/year); opioid use dose is categorized as low (0–20 MME/day), medium(20–50 MME/day), high (50–100 MME/day) and very high (>100 MME/day).

Table 2 presents results of Cox regression analysis predicting suicide attempts. Adjusting for covariates, the risk of attempts was higher among men and among those 18–25 years old. Medical comorbidities including neuropathy, chronic pain, acute pain, and cancer had significant negative associations with the risk of suicide attempts. In contrast, psychiatric disorders were strongly associated with elevated risk of attempts. A diagnosis of depression or bipolar disorder conveyed 6 times the risk of a suicide attempt (HR = 6.15, 95% CI: [5.74, 6.59]), while patients with psychotic disorders and substance use disorders had 3.63 (95% CI: [3.39, 3.95]) and 2.29 (95% CI: [2.12, 2.49]) times the risk of as attempt compared to patients without these diagnoses, respectively.

**Table 2:** Results of multivariable Cox regression for time to suicide attempts (N = 842,773).

Variable	Hazard Ratio	95% CI	p-value
**Sex**			
Men	0.916	(0.862, 0.974)	0.005
Women	1		[Reference]
**Age category**			
26–39 years	0.766	(0.691, 0.849)	<0.001
40–64 years	0.826	(0.755, 0.904)	<0.001
18–25 years	1		[Reference]
**Medical conditions**			
Headache	1.03	(0.951, 1.115)	0.473
Neuropathy	0.63	(0.552, 0.719)	<0.001
Chronic pain	0.652	(0.604, 0.705)	<0.001
Acute pain	0.863	(0.805, 0.924)	<0.001
Cancer	0.454	(0.42, 0.491)	<0.001
**Psychiatric conditions**			
Depression/Bipolar	6.147	(5.736, 6.588)	<0.001
Psychosis	3.634	(3.348, 3.945)	<0.001
Substance Use	2.294	(2.115, 2.487)	<0.001
PTSD	1.452	(1.283, 1.643)	<0.001
Anxiety	0.877	(0.813, 0.946)	0.001
**Opioid use (frequency, dose):** *			
No Use	1		[Reference]
(low, low)	0.742	(0.609, 0.904)	0.003
(low, medium)	0.531	(0.469, 0.601)	<0.001
(low, high)	0.578	(0.471, 0.709)	<0.001
(low, very high)	0.585	(0.323, 1.058)	0.076
(medium, low)	3.344	(2.866, 3.902)	<0.001
(medium, medium)	4.004	(3.618, 4.43)	<0.001
(medium, high)	4.44	(3.707, 5.317)	<0.001
(medium, very high)	2.841	(2.039, 3.96)	<0.001
(high, low)	5.177	(4.268, 6.28)	<0.001
(high, medium)	7.024	(6.182, 7.98)	<0.001
(high, high)	7.231	(6.216, 8.412)	<0.001
(high, very high)	6.712	(5.707, 7.893)	<0.001

* Opioid use frequency is categorized as low (0–1 month/year), medium (1–6 months/year), and high (6–12 months/year); opioid use dose is categorized as low (0–20 MME/day), medium(20–50 MME/day), high (50–100 MME/day) and very high (>100 MME/day).

The joint categories of opioid use frequency and dose, except low frequency coupled with a very high dose, were significantly associated with suicide attempts compared to no use. For patients with prescription opioid use of less than one month per year, the hazard ratios under 1.0 indicated that time limited and nonrecurrent opioid use was associated with a lower risk of suicide attempts compared to non-users. However, patients with 1–6 months of prescription opioid use per year at any dose had a significantly higher risk of attempts (HRs ranging from 2.8 (95% CI = 2.04, 3.96) to 4.4 (95% CI = 3.71, 5.32) compared to non-users. Similarly, for patients with more than 6 months of prescription opioid use per year, HRs for risk of suicide attempts were substantially above 1 and increased in magnitude with increasing doses. These HRs indicated that, at all dose levels, high frequency use was associated with a 5.18 (95% CI = 4.27, 6.28) to 7.2 (95% CI = 6.22, 8.41) times increased risk of an attempt relative to non-users.

**Fig 1:**
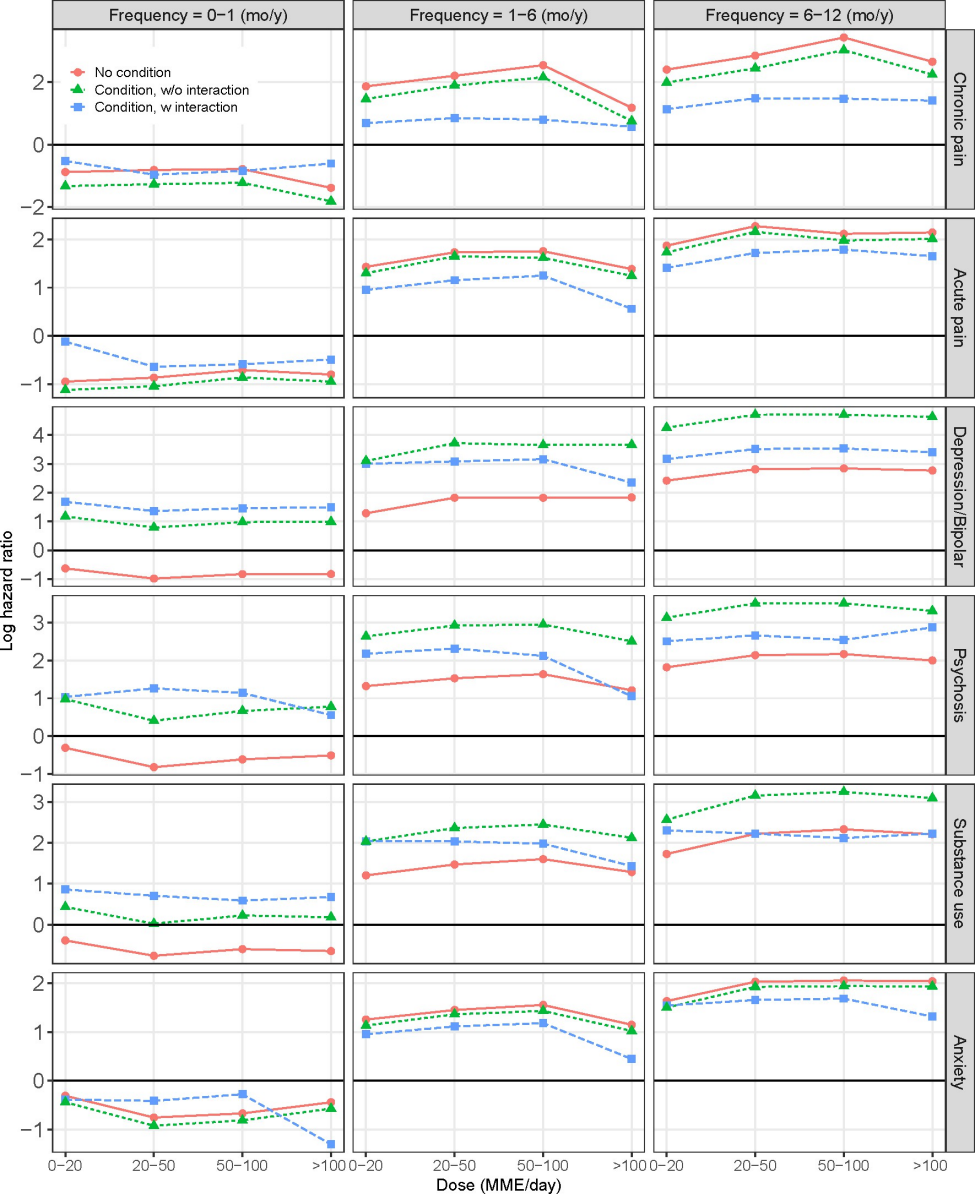
Interaction effects involving medical/psychiatric conditions and opioid use (frequency, dose) on risk of suicide attempt. Cancer, neuropathy, and PTSD were not included as their interactions with opioid use were not statistically significant. The figure is derived from models that include both main effects for opioid use and the conditions as well as the interaction term (opioid use X a particular condition). The difference between the parallel red and green lines indicates the main effect of a particular condition, while the discrepancy between the green and the blue lines indicates the interaction, i.e. the existence of medical or psychiatric conditions affects the effect of opioid usage on suicide attempt risk.

To examine the potential for differential effects of opioid use on suicide attempts based on patients’ medical and psychiatric conditions, we tested the two-way interactions involving opioid use and the conditions included in Table 2. Due to high levels of multicollinearity among these conditions, each 2-way interaction was assessed in separate models that included all main-effects from Table 2 plus the interaction term. A visualization of the patterns of statistically significant interaction effects is presented in Fig 1. In this figure, each row panel is derived from a model with the main and conditional effects of opioid use (frequency and dose) and the particular condition. The red lines show the main effects of opioid use only, the green lines show the combined main effects of opioid use and the particular condition, and the blue lines show the combined effects of opioid use and the particular condition plus the interactions between them. More detailed results are presented in S2 Fig. The difference between the parallel red and green lines indicates the main effect of a particular condition, while the discrepancy between the green and the blue lines indicates the conditional effects, i.e., the presence of medical or psychiatric conditions alters the effects of opioid usage on suicide attempts.

For depression/bipolar disorders, psychosis, and substance use disorders, the inclusion of interaction terms for these conditions and the measures of opioid use resulted in a slight downward adjustment to the increased risk due to these conditions (as indicated by the blue lines in the figure). In other words, the predicted risk of suicidal behavior derived from the fitted models containing these interaction terms was slightly lower than that derived from the combined main effects of these predictors. This downward adjustment is slight for depression/bipolar disorders, but more substantial for substance abuse disorders such that patients with more frequent and higher levels of opioid use (the righthand panel of Fig 1) had levels of suicide risk that were virtually no different than patients with this level of opioid use who did not have substance abuse disorders. In contrast, the reduced risk of suicidal behavior among patients with anxiety disorders (see main effect in Table 2) was amplified among patients with higher frequency prescription opioid use at greater dosage levels, such that higher levels/greater frequency of opioid use conveyed no additional risk of suicidal behavior among those with anxiety disorders.

Conversely, interaction effects involving prescription opioid use and pain followed a very different pattern. For patients with chronic and to a lesser extent acute pain diagnoses, compared to the main effects of the condition (red to blue lines, top panel), the large interactions result in that higher opioid frequency and dose were associated with substantially lower risk of attempts. Consider for example the contrasts among patients with chronic pain prescribed 50–100 MME/day at moderate (1–6 months) frequency in Fig 1 (red to blue lines, top middle panel). The HR of opioid use predicting suicidal behavior was reduced from 12.73 (i.e. log HR = 2.54) for those with no chronic pain to 2.22 (i.e. log HR = 0.80) for those with chronic pain. Similarly, for those at high (6–12 months) frequency use (red to blue lines, top right panel), the HR of opioid use was reduced from 30.93 (i.e. log HR = 3.43) for those with no chronic pain to 4.36 (i.e. log HR = 1.47) for those with chronic pain.

**Fig 2:**
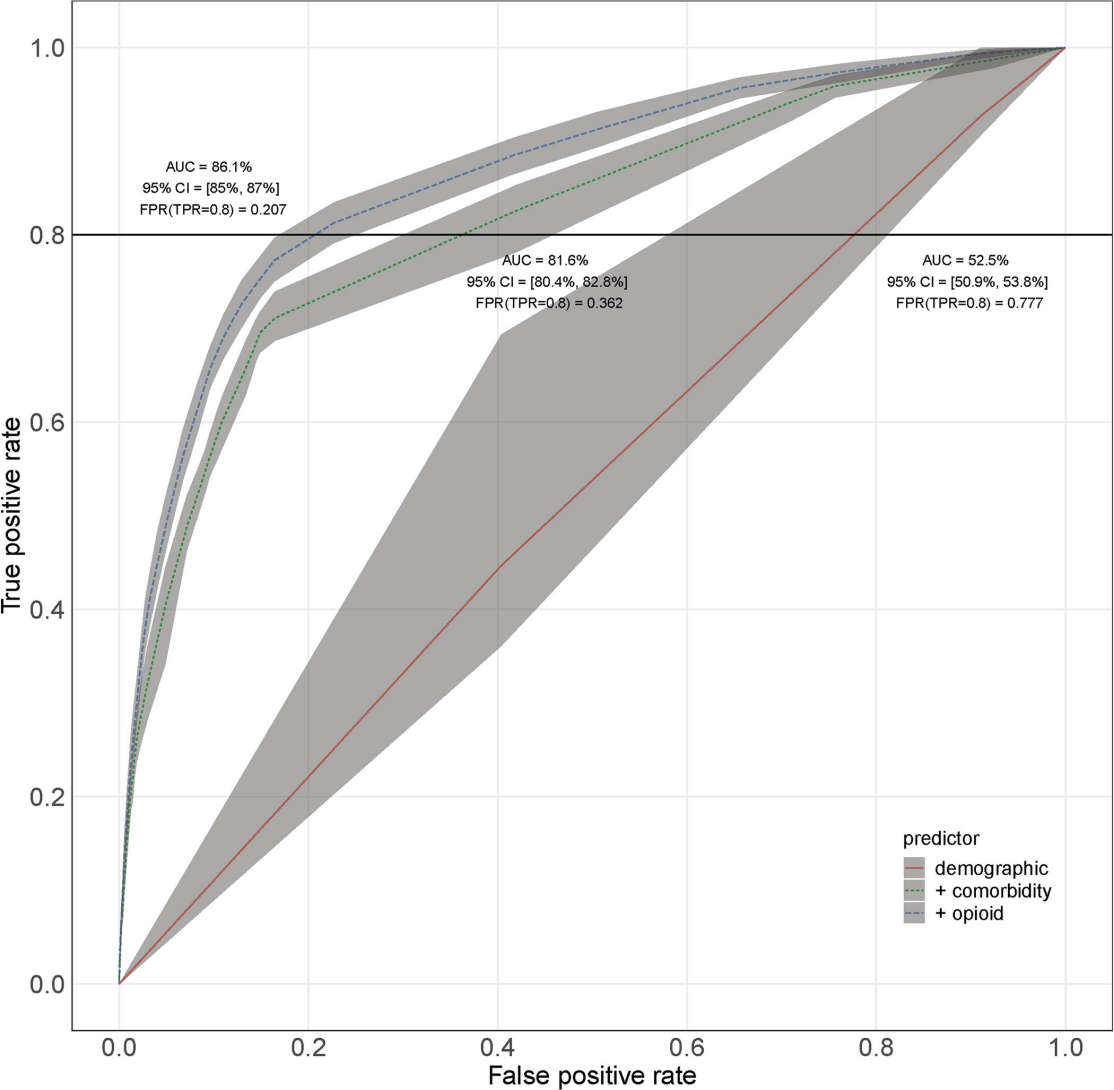
ROC curve showing the performance of one-year suicide attempt prediction using nested Cox regression models. The solid red line is from the model including only demographic variables. The dotted green line is from a model that adds the psychiatric and medical conditions, and the dashed blue line is from the model that adds the opioid use variables.

Although our primary purpose in this analysis was not to create a comprehensive model of patient suicide risk, an indication of the role of opioids in improving the quantification of patient risk is seen in Fig 2. Improvement in our ability to predict patients at risk using measures of prescription opioid use can be seen by contrasting ROC curves for suicide attempt risk among three nested models. The naïve model included only the demographic variables from Table 1 and achieved out-of-sample AUC of 52.5% (95% CI: [50.9%, 53.8%]). Adding medical and psychiatric conditions to the naive model increased the AUC to 81.6% (95% CI: [80.4%, 82.8%]), and including the main effects of the measures of opioid use in the model further increased the AUC to 86.1% (95% CI: [85.0%, 87.0%]). These results indicate that when opioid-related predictors were included in the model, the 20% of patients deemed by the model to be at highest risk captured 80% of future suicide attempts. In contrast, the model omitting opioid-related variables could only detect 80% of true positives by designating almost double this number of patients (36.2%) as at high risk for suicidal behavior.

## Discussion

This study, one of the largest and most comprehensive investigations of the association between prescription opioids and suicide to date, found a significant increase in the risk of suicidal behavior among patients with frequent opioid use, particularly at higher doses. Patients prescribed opioid medications for at least 6 months in the past year and at 20–50 MME levels or higher had approximately five to seven times the risk of attempted suicide compared to those not prescribed opioid medications. Coupled with the effects of psychiatric conditions such as depression/bipolar disorders and psychosis, frequent use of moderate to high strength prescription opioids placed such patients at substantially elevated risk of suicidal behavior. These results also highlight the potential for measures of opioid prescribing to greatly improve predictive models of suicide risk and to shrink the number of the highest risk patients for whom ongoing monitoring is warranted. This is of substantial clinical relevance with a rare outcome such as suicidal behavior, where the large size of the “haystack” creates an enormous burden for clinicians trying to find needles by surveilling at-risk patients.

Prescription opioids were not uniformly associated with increased risk of suicidal behavior, however. For patients with chronic pain, higher rates of suicidal behavior were observed among those *not* consistently taking prescription opioids, suggesting an additional rationale for ensuring adequate pain management in these patients. This finding highlights the complex clinical challenge involved in balancing appropriate pain management, mitigating the risk of opioid misuse [31], and reducing the risk of suicidal behavior. In addition, suicide risk was lower among individuals with anxiety disorders other than PTSD, which may indicate that the sedative effects of opioids modulate anxiety-induced suicidal behavior. Patients in pain often experience pain-related anxiety which can introduce new anxiety or be additive to an existing anxiety history [32,33]. However, the relationship between prescription opioid medications and anxiety is complex, with evidence that opioids are associated with both improving and worsening of anxiety symptoms [34].

From a policy perspective our findings have direct relevance to prescription drug monitoring programs, one of the nation’s most prominent strategies for curtailing opioid misuse and injury. Recent studies in New York and Florida indicate that state-based prescribing restrictions can result in modest reductions in the frequency, intensity, and duration of opioid use [35,36]. Such restrictions, however, are a blunt instrument, and concerns have been raised about the potential for undertreatment of pain [37,38]. Our results demonstrate the tension associated with reducing opioid prescribing and appropriate management of pain and other comorbid conditions as it relates to suicide risk. While frequent use and/or high doses of prescription opioids was associated with roughly 5 to 7 times the risk of suicide attempt in our study, this was only true of patients who ***did not*** have a medical diagnosis where pain management can be challenging. The heightened suicide risk of such patients in the absence of opioid medications highlights the pressing need for a better understanding of the costs and benefits of restricting prescription opioid use, particularly in the context of worsening mental health and increased risk of suicide.

This study has a number of limitations. It is restricted to commercially insured patients that received their insurance through the state of Connecticut or through fully insured health plans, which constitutes approximately 35–40% of commercial patients in the state. Replication and expansion of these analyses with more recent data and with patients covered by public insurance is warranted, particularly given evidence of the medical burden associated with opioid use disorders among Medicaid patients [39]. Second, it uses claims data that capture prescription “fills” but does not take into account opioids obtained through other means and actual patient opioid use. Third, our data did not allow us to examine non-medical prescription opioid use, self-medication with opioids for depressive and other mental disorders, as well as the relationship between illicit use of (prescription and non-prescription) opioids and suicidal behavior, all of which may complicate and confound the observed associations among prescription opioid use, mental disorders, and suicide attempts [40–43]. We also do not know whether these patients’ psychiatric or medical symptoms were well controlled which could influence the relationship between opioid prescribing and risk of suicide. Fourth, a further limitation of the study is that our data and methodology do not allow us to infer causality. Finally, we did not have data on suicide mortality; based on Connecticut death data this could have changed the status for approximately 100–200 patients on measures of suicide risk.

## Conclusion

Our findings reveal a complex relationship between prescription opioids, mental health, pain and other medical comorbidities, and suicide attempts, such that prescription opioids may be associated with both increased, and decreased, suicide risk. Such findings highlight the need for clinicians to give added clinical attention in the prescribing and monitoring of prescription opioids in patients with psychiatric disorders. In addition, these findings indicate that a more nuanced approach may be needed to manage and monitor patients’ access to such medications, one that is patient-centered and explicitly informed by health risks other than the potential for opioid misuse and abuse.

## Supporting information

S1 Fig Study design. Description of the study design using four hypothetical patients. The first three patients are included because they have medical claims during the recruitment window. All three are observed till their first suicide attempt or December 31, 2015. The fourth patient is not included in the study as there were no medical records in the recruiting window. The opioid use doses of the first patient are shown to demonstrate the calculation of the opioid features. This patient had the first medical record during the recruiting window at month 2 and had suicide attempt at month 41. Eight opioid prescriptions are identified at months 6, 9, 30, 37, 38, 39, 40, 41 with monthly MME 30, 20, 10, 50, 50, 40, 75, 65 respectively. During the patient’s 40-months observation period, the opioid frequency is 8 / 40 * 12 = 2.4 months per year, the median MME is 45. During the patient’s last 6 months of observation period the opioid frequency is 5/6*12 = 10 month/year and the median MME is 50, thus the trend of frequency and MME are both increasing.
(TIF)

S2 Fig The interaction effects involving each psychiatric and medical condition with opioid use in predicting suicide attempts. The numbers in each cell are log hazard ratio (row 1), likelihood ratio (row 2), p-value of the likelihood ratio test (row 3). Those with statistically significant interaction effects (p<0.0004 after Bonferroni corrections adjusting for multiple testing) are marked with red (positive interaction) or purple (negative interaction) color.
(TIF)

S1 Table ICD-9 code for suicide attempt. (DOCX)

S2 Table ICD-9 codes of the comorbidities from the Veteran’s suicide study [13]. (DOCX)
