# Supplementary figures and images for: The association of prescription opioid use with suicide attempts: An analysis of statewide medical claims data
Source: PLoS One. 2022 Jun 30;17(6):e0269809. doi: 10.1371/journal.pone.0269809 (PMC9246186; doi:10.1371/journal.pone.0269809)

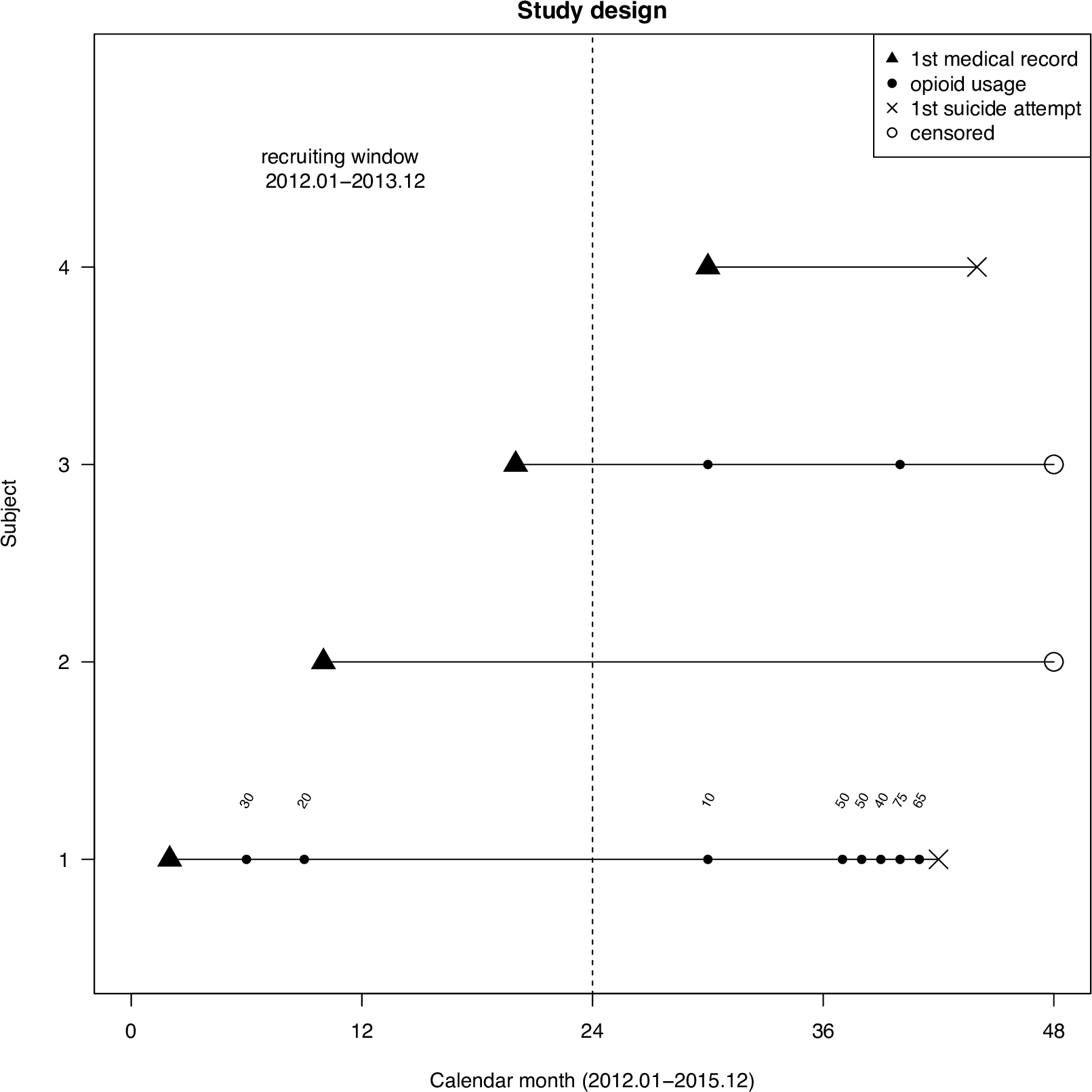

Supplement: S1 Fig — Description of the study design using four hypothetical patients. The first three patients are included because they have medical claims during the recruitment window. All three are observed till their first suicide attempt or December 31, 2015. The fourth patient is not included in the study as there were no medical records in the recruiting window. The opioid use doses of the first patient are shown to demonstrate the calculation of the opioid features. This patient had the first medical record during the recruiting window at month 2 and had suicide attempt at month 41. Eight opioid prescriptions are identified at months 6, 9, 30, 37, 38, 39, 40, 41 with monthly MME 30, 20, 10, 50, 50, 40, 75, 65 respectively. During the patient’s 40-months observation period, the opioid frequency is 8 / 40 * 12 = 2.4 months per year, the median MME is 45. During the patient’s last 6 months of observation period the opioid frequency is 5/6*12 = 10 month/year and the median MME is 50, thus the trend of frequency and MME are both increasing. (TIF) [file pone.0269809.s001.tif]

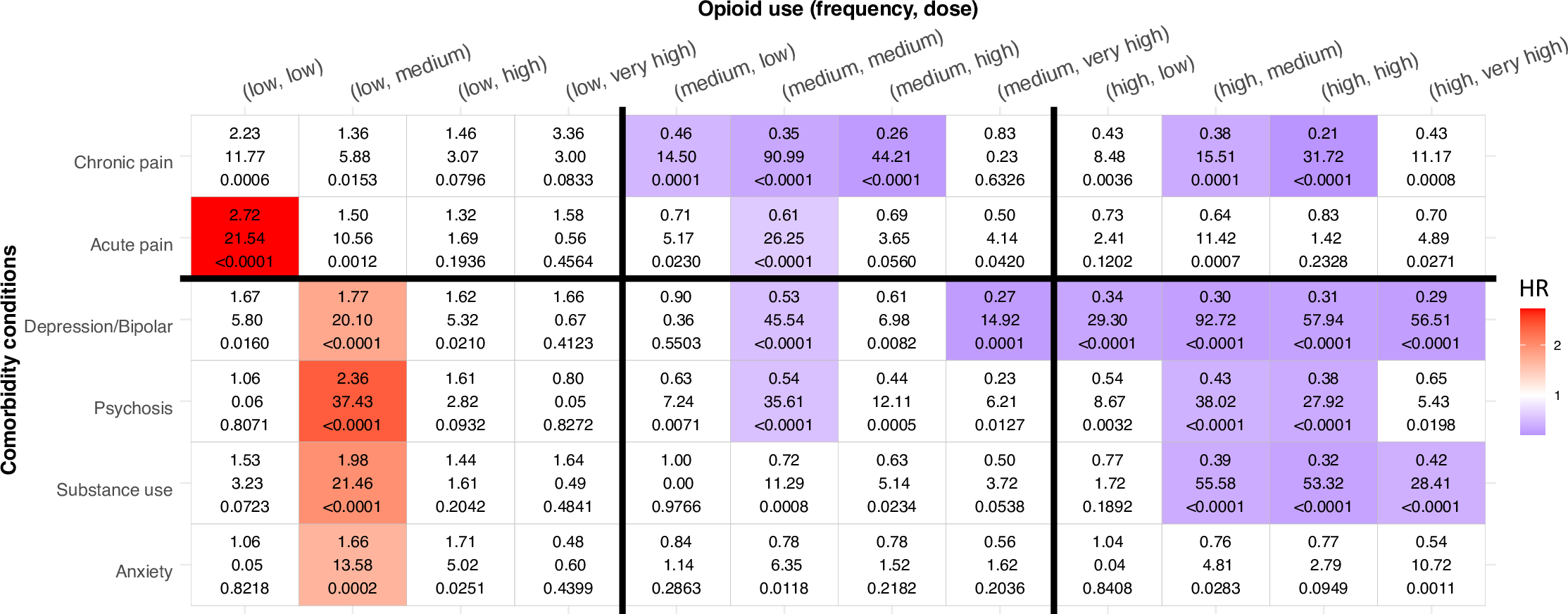

Supplement: S2 Fig — The numbers in each cell are log hazard ratio (row 1), likelihood ratio (row 2), p-value of the likelihood ratio test (row 3). Those with statistically significant interaction effects (p<0.0004 after Bonferroni corrections adjusting for multiple testing) are marked with red (positive interaction) or purple (negative interaction) color. (TIF) [file pone.0269809.s002.tif]
