# Supplementary material for: The association of prescription opioid use with suicide attempts: An analysis of statewide medical claims data
Source: PLoS One. 2022 Jun 30;17(6):e0269809. doi: 10.1371/journal.pone.0269809 (PMC9246186; doi:10.1371/journal.pone.0269809)
Supplement: S1 Table — (DOCX) [file pone.0269809.s003.docx]

**Supplemental Table 1****: ICD-9 code for suicide attempt.**

| **Code Type** | **ICD-9 codes** |
| --- | --- |
| **Suicide Attempt** | E950-E958 (intentional self-harm) |
| **Suicide V-Code** | V62.84 (suicide ideation) --AND SAME VISIT-- 870-899, 960-989 |
| **Suicide Algorithm** | 881, 960-979, 980-989, 994.7 ----AND SAME VISIT---- 293.83, 296.20-296.36, 296.82, 296.90, 298.0, 300.4, 309.0-309.1, 311, 296.00-296.06, 296.1-296.14, 296.40-296.89, 296.99, 301.13, 301, 290.8-290.9, 295, 297, 298.1-298.9, 299, 301.20-301.22, 780.1, 309.2-309.9 |
