# Supplementary material for: The association of prescription opioid use with suicide attempts: An analysis of statewide medical claims data
Source: PLoS One. 2022 Jun 30;17(6):e0269809. doi: 10.1371/journal.pone.0269809 (PMC9246186; doi:10.1371/journal.pone.0269809)
Supplement: S2 Table — (DOCX) [file pone.0269809.s004.docx]

**Supplement table 2. ICD-9 codes of the comorbidities from the Veteran’s Suicide Study [13].**

| **Comorbidity** | **ICD-9 codes** |
| --- | --- |
| Headache | 339, 346, 307.81, 784.0, 350.2 |
| Neuropathy | 337.0-337.1, 355-357, 377 |
| Chronic pain^1^ | 338.2[1289], 338.4, 346, 307.81, 71x, 72x |
| Acute pain^2^ | 282.62, 338. 1[1289], 522.[57], 57[47], 592, 733.1, 8[012367]x, 84[0-8], 85[0-4], 8[89][0-7], 90[0-4], 9[1-5] |
| Cancer^3^ | 140-172, 174-239, 3383 |
| Depression,  Bipolar disorder,  Mood disorder NOS | 293.83, 296.2-296.3, 296.90-296.99, 298.0, 300.4, 301.12, 309.0-309.1, 311, 296.0–296.1, 296.4–296.8 |
| Psychotic  Disorders | 295.0-295.4, 295.6-295.9, 297.0-297.3, 297.8- 298.4, 298.8, 298.9 |
| Substance  Use disorders^4^ | 291-292, 303.0, 303.9, 304.1–305.0, 305.2-305.4, 305.6-305.9 |
| PTSD | 309.81 |
| Anxiety | 300.00-300.02, 300.09-300.10, 300.20–300.23, 300.29 |
| 1. CDC value set [15]. 338.2[1289] means 338.21, 338.22, 338.28, 338.29 2. CDC value set. 3. Excludes 173xx (non-melanoma skin cancers) 4. Excludes those subjects with opioid abuse disorder (3040, 3055)**.** | |
